# miR-22 protect PC12 from ischemia/reperfusion-induced injury by targeting p53 upregulated modulator of apoptosis (PUMA)

**DOI:** 10.1080/21655979.2020.1729321

**Published:** 2020-02-15

**Authors:** Hongmei Jiao, Renyi Chen, Ziru Jiang, Lin Zhang, Hongwei Wang

**Affiliations:** a Department of Anesthesia, Linyi Cancer Hospital, Linyi, Shandong, China; b External Abdominal Section, Linyi Cancer Hospital, Linyi, Shandong, China

**Keywords:** Ischemia/reperfusion, cerebrovascular diseases, neuroprotecti﻿ve, apoptosis, microRNA, p53 upregulated modulator of apoptosis

## Abstract

MicroRNAs have been implicated as critical regulatory molecules in many cerebrovascular diseases. Recent studies demonstrated miR-22 might provide a potential neuroprotective effect. However, the neuroprotective effect of miR-22 in ischemia/reperfusion (I/R) injury has not been thoroughly elucidated. In this study, the PC12 cells were subjected to 4 h oxygen and glucose deprivation (I) and 24 h reoxygenation (R). The PC12 cells were pre-transfected with miR-22 or anti-miR-22 or siRNA-mediated downregulation of p53-upregulated-modulator-of-apoptosis (PUMA)(PUMA siRNA) or their controls at 24 h prior to exposure to I/R. Reverse transcription-quantitative polymerase chain reaction (RT-qPCR) and Western blot were employed to analyze mRNA and protein expression. PI and Annexin V assays and terminal deoxynucleotidyltransferase-mediated dUTP-biotin nick end labeling (TUNEL) assay were used to quantify the rate of apoptosis. We found that miR-22 expression was significantly downregulated in the PC12 cells subjected to I/R. Loss of function of miR-22 increased PC12 apoptosis after I/R, and overexpression of miR-22 decreases PC12 apoptosis after I/R. PUMA protein was upregulated in the I/R group as compared with the sham group. The increased PUMA protein expression and apoptosis induced by I/R was reversed by transfection with PUMA siRNA. We concluded that I/R enhanced apoptosis and PUMA expression in PC12 cells via downregulation of miR-22. Enhanced miR-22 expression reversed both PUMA expression and apoptosis induced by I/R in PC12 cells. miR-22/PUMA axis has important implications for their clinical applications.

## Graphical Abstract

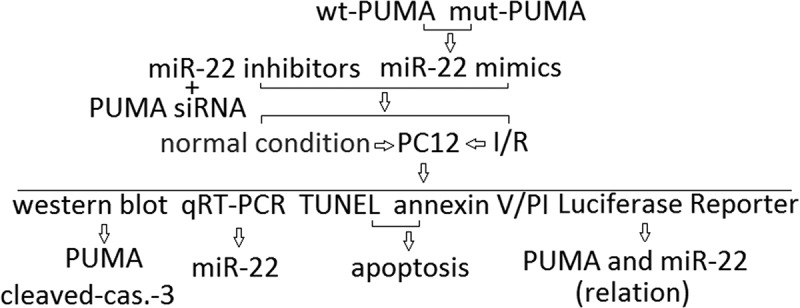

## Introduction

Cerebral ischemia is a serious condition associated with vascular disease, affecting patients worldwide. Despite hundreds of promising preclinical trials demonstrating efficacy of neuron-targeted therapies in animal models of stroke, the only clinical treatment remains early restoration of blood flow with thrombolysis [1]. The failure to translate neuron-targeted approaches to useful clinical therapy suggests that alternative cellular targets in the brain may more effectively coordinate the complex intra- and intercellular signaling cascades that contribute to neuronal injury. Although the apoptosis is a prominent cellular injury mechanism, understanding the mechanisms underlying cerebral neuron apoptosis is still the key prerequisite for the treatment of brain ischemia/reperfusion (I/R) injuries effectively [2,3].

MiRs are a class of small noncoding RNA molecules (_~_22 nts) found in most eukaryotes, including humans [4]. Its discovery has largely broadened our understanding of the mechanisms that regulate gene expression in various physiological and pathological conditions. It is known that miRNAs can induce translational silencing and repress target protein production through binding to the 3′-UTR region of the target mRNA [5]. The brain, of all other mammalian organs, expresses the highest number of microRNAs (miRs) [6] and therefore they have been considered as essential modulators of many brain functions in both physiological and pathological conditions [7–10]. In glia, miRs have been shown to play an important role in the cellular response to ischemic injury. In particular, miRs can alter the expression of proteins that both directly and indirectly modulate glial mitochondrial function [9,10]. miR-22-5p (miR-22) belongs to a small non-coding RNA family and functions in the gene silencing and post-transcriptional regulation of mRNA. Growing evidence supports that miR-22 is involved in multiple cellular biological processes, such as organ or cell ischemia/reperfusion injury. Yang et al. reported that adenovirus-mediated miR-22 overexpression markedly reduced the release of creatine kinase and lactate dehydrogenase, infarct size and cardiomyocytes apoptosis [11]. However, in H9c2 cardiomyocyte, it was found that miR-22 overexpression aggravated, whilst miR-22 inhibitor significantly attenuated H/R-induced mitochondrial damage [12]. Li et al. reported that compared with the sham group, miR-22 was upregulated in a model of spinal cord ischemia in Sprague Dawley rats but downregulated during reperfusion [13]. However, wang et al. reported that miR-22 mRNA level in the brain tissue of cerebral ischemia–vreperfusion rats was significantly decreased, and enforced miR-22 expression exerted the neuroprotective effect via the PI3K/Akt signaling pathway [14]. Yu et al. reported that miR-22 overexpression resulted in decreased cerebral I/R injury followed by reduced inflammatory cytokines and cortical neurons apoptosis [15]. The data indicated that miR-22 exerts different functions and mechanisms in I/R-induced injury in different organs and cells.

PUMA (p53 upregulated modulator of apoptosis) is a pro-apoptotic member of the BH3-only subgroup of the Bcl-2 family. PUMA inhibits all the five anti-apoptotic proteins (Mcl-1, Bcl-2, Bcl-XL, Bcl-W and A1) and directly triggers apoptosis mediated by pro-apoptotic proteins Bax/Bak [16]. Recently it has found that targeting lncRNA myocardial infarction-associated transcript (MIAT) protects against H9c2 cardiomyoblasts H/R injury or myocardial I/R injury via inhibition of cell apoptosis, mediated by NF-κB and PUMA signal pathway [17]. PUMA is an essential mediator of cardiomyocyte death upon I/R injury and offers therapeutic target to limit cell loss in ischemic heart disease via inhibiting cell apoptosis [18]. Wu et al. reported that PUMA is activated by oxidative stress in response to I/R to promote p53 independent apoptosis in the small intestine through the mitochondrial pathway, and Inhibition of PUMA protected against I/R-induced intestinal injury and apoptosis [19]. Wang et al. reported that suppressing p53/Puma-mediated mitochondrial caspase-9/3 apoptosis pathway protected OGD/R or I/R-induced injury in PC12 cells and cerebral ischemia [20]. Therefore, regulating the levels and function of PUMA is expected to become a new therapeutic target against I/R.

Recent studies have shown that few miRNAs can regulate PUMA activity directly or indirectly. For example, miR-29a mimic protects and miR-29a inhibitor aggravates cell injury and mitochondrial function after ischemia-like stresses in vitro via regulating PUMA expression [21]. In renal cell carcinoma, enforced overexpression of miR-22 in renal cancer cells inhibited proliferation and induced cell apoptosis via activating p53 and its downstream target p21 and PUMA, and the apoptosis markers cleaved CASP3 and PARP [22]. miR-22 was downregulated and PUMA was upregulated in cerebral I/R injury [15,20]. In the present study, we investigated the role and mechanisms of miR-22 in PC12 cells subjected to I/R. We found that miR-22 was downregulated in PC12 cells subjected to I/R. Enforced miR-22 was able to repress pro-apoptotic target PUMA and attenuate cell apoptosis, respectively, thus contributing to PC12 cell protection induced by I/R injury. Therefore, miR-22/PUMA signals may provide a new insight to develop an effective pharmacological or genetic agent aimed at the molecular target.

### Materials and methods

#### Cell culture

Rat pheochromocytoma-derived cell line (PC12) was obtained from American Type Culture Collection (ATCC, Shanghai, China) and maintained at 37°C in a humidified atmosphere (90%) containing 5% CO_2_. Cells were cultured in Dulbecco’s modified Eagle’s medium (DMEM) with 10% (v/v) heat-inactivated FBS, 100 Uml−1 penicillin, and 100 mg/ml−1 streptomycin.

#### miR-22 mimics and inhibitors transfection

To inhibit the expression of miR-22 in PC12, we transfected mirVanna miRNA inhibitors (Life Technologies) specific to miR-22 (anti- miR-22) or a miR inhibitor negative control (anti-miR-NC) using Lipofectamine 2000 reagent (Invitrogen) as the manufacture’s instruction. For gain-of-function studies, we transfected the cells with a miR mimic negative control (miR-NC), miR-22 mirVanna mimics (miR-22) (Life Technologies). Transfected cells were incubated overnight in serum-free medium supplemented with 0.1% BSA, 10 mM 4-(2-hydroxyethyl)-1-piperazineethanesulfonic acid (HEPES) (pH 7.4), and 1% penicillin. To block the PUMA expression, the PUMA siRNA or control siRNA (Sigma-Aldrich) was transfected with PC12 cells for 8 h before I/R.

The miR-22 mimics, inhibitor, NC were designed and subsequently synthesized by Life Technologies. The hsa-miR-22-3p mimic sequences (named miR-22 mimic): sense: 5′-AAGCUGCCAGUUGAAGAACUGU-3′, and the negative control duplex (miR-NC): sense: 5′-ACUACUGAGUGACAGUAGA-3′.miR-22 inhibitor (anti-miR-22): 5′-ACAGUUCUUCAACUGGCAGCUU-3′; anti-miR- NC: 5′-CAGUACUUUUGUGUAGUACAA-3′. The siRNA sequences (sense strands) against PUMA: 5′-GAGCGGCGGAGACAAGAAGA GUU-3′; and control sense, 5′-ACUUAACCGGCAUACCGGC-3′, were purchased from Thermo Fisher Scientific. All in vitro assays were determined 0–48 h after transfection when maximum knockdown efficiency was reached.

#### Luciferase reporter assay

Wild-type (wt) or mutant (mut) of 3ʹ-UTR of PUMA were inserted into pGL3-Basic vector (Promega, Madison, Wisconsin) and named as wt-PUMA or mut-PUMA. After 24 h of incubation, the cells were co-transfected with wt-PUMA or mut-PUMA (500 ng) and miR-22 mimic or miR-NC (50 nM) using Lipofectamine 2000. Twenty-four hours after transfection, luciferase activity was measured using dual-luciferase reporter assay (Promega) with Renilla luciferase as internal control.

#### In vitro *simulated I/R*

PC12 plated on coverslips or six-well plates were transfected with miR inhibitors or miR mimics, washed with 1 × PBS, and placed in an ischemia buffer that contained (in mM) 118.0 NaCl, 24.0 NaH2CO3, 1.0 NaHPO4, 2.5 CaCl_2_, 1.2 MgCl_2_, 20.0 sodium lactate, 16.0 KCl, and 10.0 2-deoxyglucose (pH 6.2). PC12 cells were then incubated in an anoxic chamber (5% CO_2_-0% O_2_) for 1 h followed by replacement of the ischemic buffer with normal cell medium and were incubated under normoxic conditions for 4 h to complete the IR (I/R) protocol as described [23]. Coverslips or plates were processed for qRT-PCR, Western blot and apoptosis assay.

#### RNA isolation and quantitative real-time RT-PCR

TaqMan MicroRNA Assays (Applied Biosystems, South San Francisco, CA, USA) were used to quantify the levels of mature miRNAs, following the manufacturer’s instructions. The miRNAs were isolated from cells using the mirVana miRNA isolation kit (Applied Biosystems), and the specific primers for detecting miR-22 were purchased from Applied Biosystems. Reverse transcription was determined using the TaqMan microRNA reverse transcription kit (Applied Biosystems) according to the manufacturer’s instructions. U6 snRNA (U6) was as an endogenous control. The following reaction components were used for the probe: 1.33 μl cDNA, 10.00 μl 2 × TaqMan Universal PCR Master Mix (Life Technologies), 1.00 μl probe, and 7.67 μl water in a 20.00-μl total volume. Each TaqMan Assay was conducted in triplicate.

#### Western blot assay

The cells were lysed in RIPA buffer (Sigma). The proteins were separated using SDS-polyacrylamide gel electrophoresis and were transferred to nitrocellulose membranes. The membranes were blocked using 5% skim milk in Tris-buffered saline containing Tween and incubated with primary antibodies directed against PUMA, cleaved-caspase-3 (anti-rabbit polyclonal antibody, Cell Signaling Technology) or β-actin (anti-mouse polyclonal antibody, Sigma) overnight at 4°C. The membranes were then incubated with horseradish peroxidase-conjugated secondary antibodies (Santa Cruz Biotechnology). The blotted membranes were visualized using enhanced chemiluminescence reagents (GE Healthcare). Western blot quantification was determined using ImageJ software.

#### PI and Annexin V assays for apoptosis detection

After *in vitro* I/R treatment, PC12 cells were collected in 48-h reperfusion time points and then washed with PBS twice. Following centrifugation, the PBS was discarded and the cells were stained with Annexin V-FITC and PI staining kit (BD Biosciences) for 15 min at room temperature in the dark. Flow cytometric analysis was determined using the Becton Dickinson FACSCalibur cytometer. Both early (Annexin V-positive, PI-negative) apoptotic cells and late (Annexin V-positive and PI-positive) apoptotic cells were analyzed. Totally, 10,000 cells were analyzed per sample.

#### Cell apoptosis by TUNEL staining

DNA fragmentation was detected in situ using terminal deoxynucleotidyltransferase-mediated dUTP-biotin nick end labeling (TUNEL) assay as the previous report [24]. In brief, PC12 cells were incubated with proteinase K, and DNA fragments were labeled with fluorescein-conjugated dUTP using terminal deoxynucleotidyl transferase (Roche Diagnostics). The total number of nuclei was determined by manual counting of 4ʹ, 6-diamidino-2-phenylindole (DAPI)-stained nuclei in six random fields per coverslip (original magnification, ×200). All TUNEL-positive nuclei were counted in each coverslip. Digital photographs of fluorescence were acquired with a Zeiss microscope (ApoTome.2; Carl Zeiss) and processed with Adobe Photoshop.

### Statistical analysis

Statistical analysis was conducted using SPSS22.0 software. The difference between groups was analyzed using unpaired or paired Student’s *t*-test when there were only two groups or assessed by one-way ANOVA followed by the Tukey’s multiple comparison tests when there were more than two groups. All tests carried out were two-tailed. Differences were considered as significant when *p* < 0.05.

## Results

### miR-22 was downregulated in PC12 subjected to I/R

**Figure 1.**
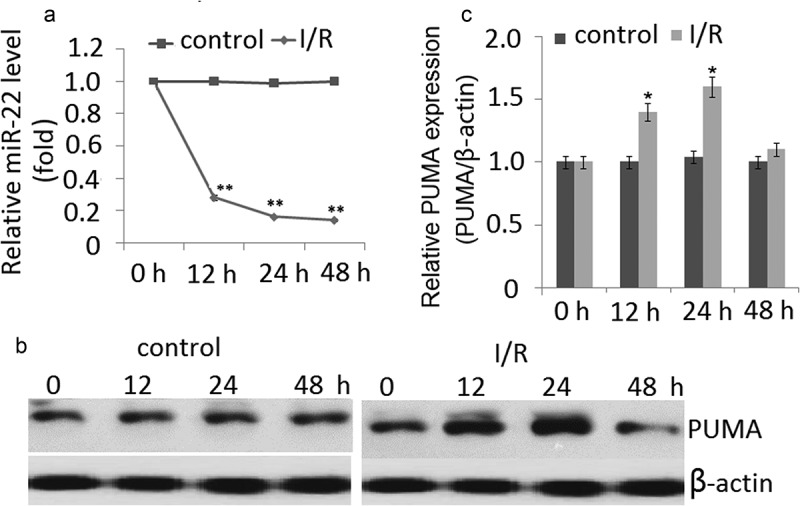
Expression of miR-22 and PUMA in PC12 cells subjected to I/R. (a) miR-22 was detected by qRT-PCR in PC12 cells subjected to I/R for 0–48 h. (b) Representative Western blot of PUMA in control and I/R groups. (c) The semiquantitative analysis of PUMA protein in PC12 cells at reperfusion 48 h after ischemia period. Data are presented as ±SD. *p < 0.05,**p < 0.01.

To investigate the potential role of miR-22 involved in I/R injury, quantitative real-time PCR was used to detect the expression levels of miR-22 in PC12 cells subjected to I/R. As shown in Figure 1(a), the miR-22 expression was significantly downregulated in PC12 cells subjected to I/R at 12 h, and reached the lowest level at 48 h. There was a sixfold decrease compared to the normal condition groups at 48 h (control) (*P* < 0.01). No significant change of miR-22 expression was observed in the normal condition groups (Figure 1(a)).

#### PUMA was upregulated in PC12 cells subjected to I/R

As shown in Figure 1(b,c), under normal condition, no significant change of PUMA expression was found in the PC12 cells during 0, 12, 24 and 48 h. However, a significant increase in PUMA protein expression was observed at the I/R-12 h compared to the normal condition group. The expression of PUMA further increased and reached a peak at 24 h (*p* < 0.01). But PUMA expression was significantly decreased at the I/R- 48 h. No significant change of miR-22 expression was observed in the normal condition groups (Figure 1(b,c)).

#### miR-22 directly regulates proapoptotic PUMA

**Figure 2.**
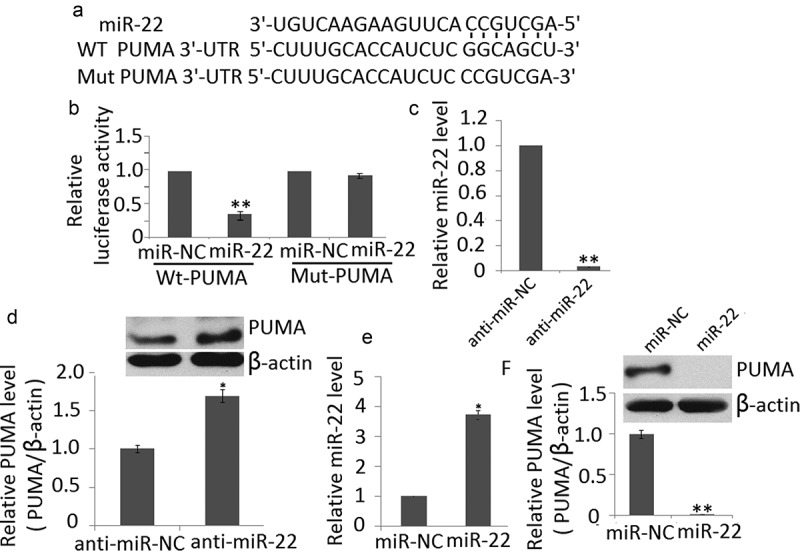
PUMA is target of miR-22. (a) PUMA 3′ UTR contains one predicted miR-22 binding site. The alignment of the seed regions of miR-22 with PUMA 3′ UTR is shown (b) miR-145 mimic transfection inhibits the luciferase activity of PC12 cells transfected with wt-PUMA but not mut-PUMA. (c) anti-miR-22 transfection inhibited miR-22 expression in PC12 cells by qRT-PCR (d) miR-22 downregulation increased endogenous levels of PUMA protein in PC12 cells (e) miR-22 transfection increased miR-22 expression in PC12 cells by qRT-PCR (f) miR-22 upregulation decreases endogenous levels of PUMA protein in PC12 cells. Data are presented as ±SD. **p < 0.01; *p < 0.05.

We determined a bioinformatics search (TargetScan, Pictar, RNhybrid) for putative mRNA targets of miR-22. Among the candidate targets, we selected 3′ UTR of human PUMA, which contained a region that matched the seed sequences of miR-22 (Figure 2(a)). Luciferase activity reporter assay revealed that miR-22 mimic transfection significantly reduced luciferase activity of PC12cell lines transfected with wt-PUMA but not mut-PUMA (Figure 2(b)).

To determine if miR-22 could affect PUMA expression in cell lines, we downregulated miR-22 in PC12 cells by anti-miR-22 transfection. Decreased expression of this miRNA on transfection was confirmed by qRT-PCR (Figure 2(c)). miR-22 downregulation significantly increased the endogenous levels of PUMA at protein levels compared with PC12 cells transfected with the anti-miR (Figure 2(d)). We also found that miR-22 upregulation (Figure 2(e)) significantly decreased the endogenous levels of PUMA at protein levels compared with PC12 cells transfected with the miR-NC (Figure 2(f)). Taken together, these results indicate that PUMA is a direct target of miR-22.

#### Apoptosis was induced in PC12 subjected to I/R

**Figure 3.**
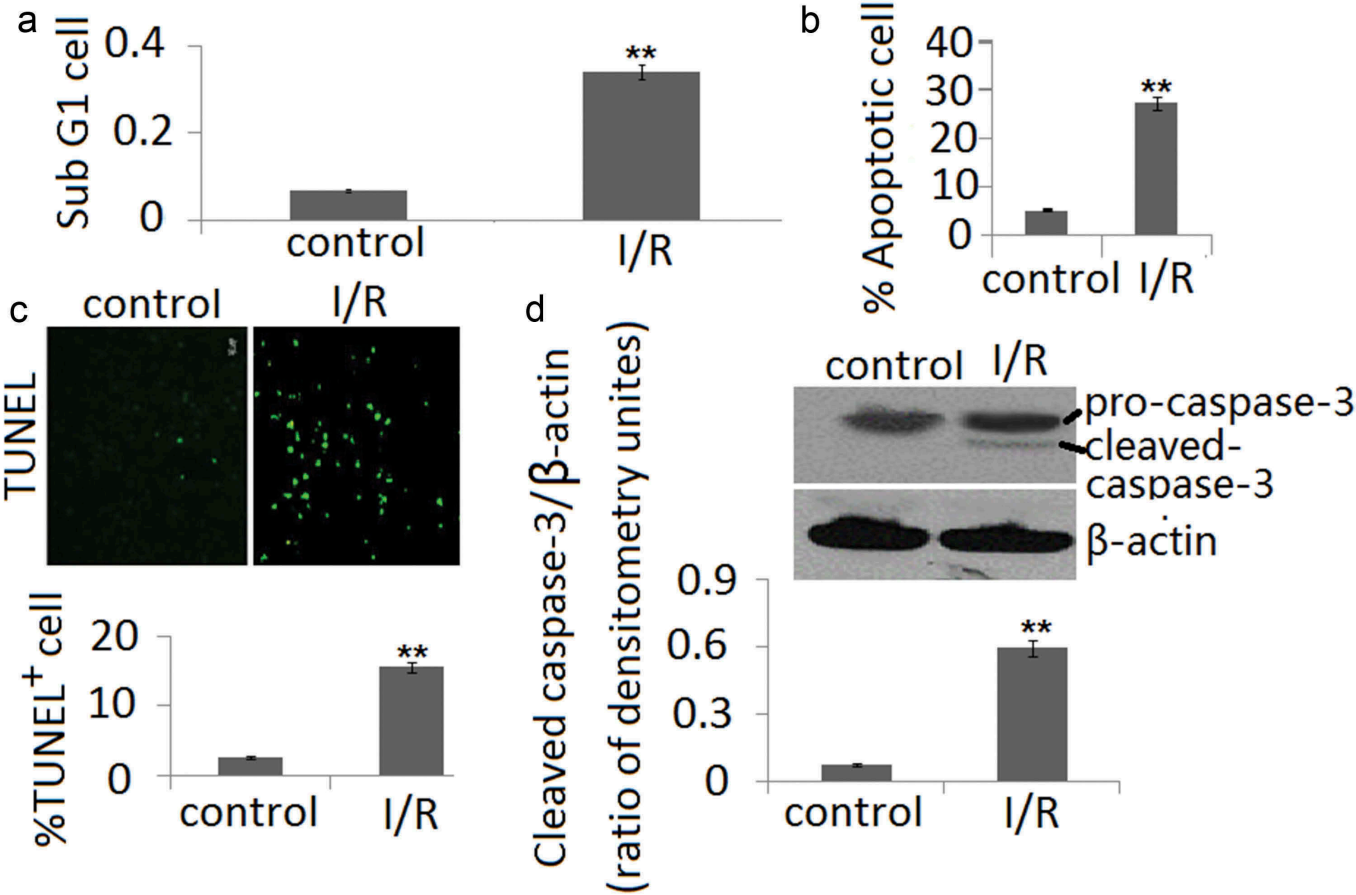
Cell apoptosis in PC12 cells during in vitro I/R. (a) Percentages of cells with the hypodiploid DNA content (sub-G1 cells) were determined by flow cytometry. (b) Cell apoptosis was determined by Annexin V and PI dual staining and determined by flow cytometry. (c) Cell apoptosis was determined by TUNEL staining. (d). The protein expression of cleaved-caspase-3 form was determined by Western blotting. The ratio of densitometry units of cleaved caspase-3/β-actin was represented in I/R and control groups. Data are presented as the mean ± SD in three independent experiments. **P < 0.05 as compared with the vehicle control group.

As shown in Figure 3(a), the sub-G1 PC12 cell percentages at reperfusion 48 h after ischemia period were obviously higher than the controls. Furthermore, the annexin V/PI staining showed that the apoptotic cells are markedly increased during I/R (Figure 3(b)). TUNEL staining of PC12 cell in vitro in conjunction with I/R showed similar results as annexin V/PI staining (Figure 3(c)). The increase of caspase-3 cleavage was also shown in PC12 cells during I/R (Figure 3(d)).

#### miR-22 protects PC12 against apoptosis

**Figure 4.**
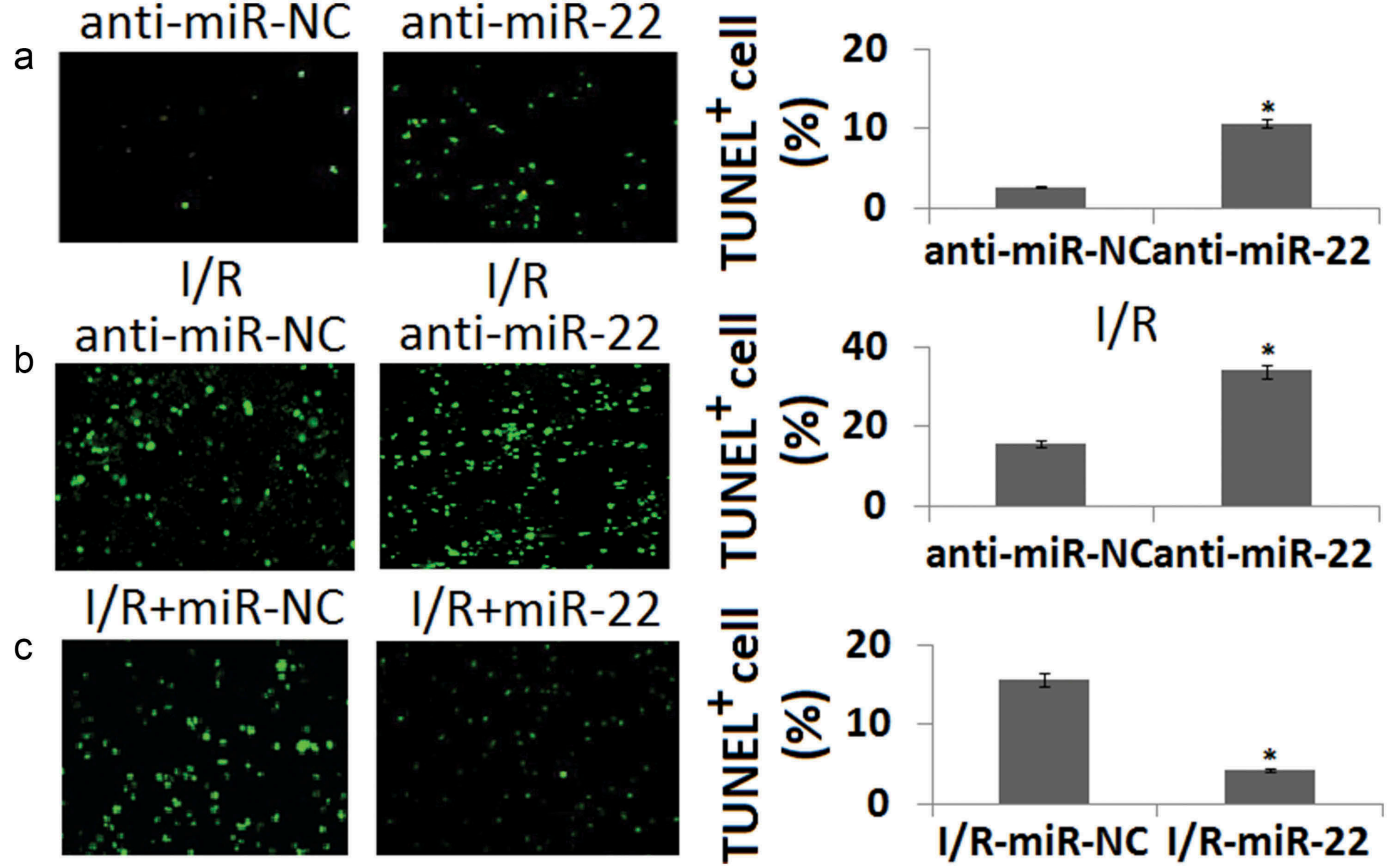
Cell apoptosis in PC12 cells transfected with anti-miR-22 or miR-22 during in vitro I/R. PC12 was transfected with anti-miR-NC or anti-miR-22 for 48 h. Cell apoptosis was determined by TUNEL staining. (b) PC12 was transfected with anti-miR-NC or anti-miR-22 followed by in vitro I/R 24 h. Cell apoptosis was determined by TUNEL staining. (c) PC12 was transfected with miR-NC or miR-22 followed by in vitro I/R 24 h. Cell apoptosis was determined by TUNEL staining. **P < 0.05 as compared with the vehicle control group.

To determine the importance of miR-22 for PC12 survival, we determined TUNEL staining of PC12 in vitro in conjunction with I/R. Loss of function of miR-22 in PC cells increased PC12 cell apoptosis under both basal conditions compared to the anti-miR-NC groups (P < 0.05, Figure 4(a)) and following I/R (*P* < 0.05, Figure 4(b)). However, enforced miR-22 expression decreased PC12 cell apoptosis under the conditions following I/R compared to the miR-NC groups (P < 0.05, Figure 4(c)).

#### miR-22 protects PC12 against apoptosis via targeting PUMA

**Figure 5.**
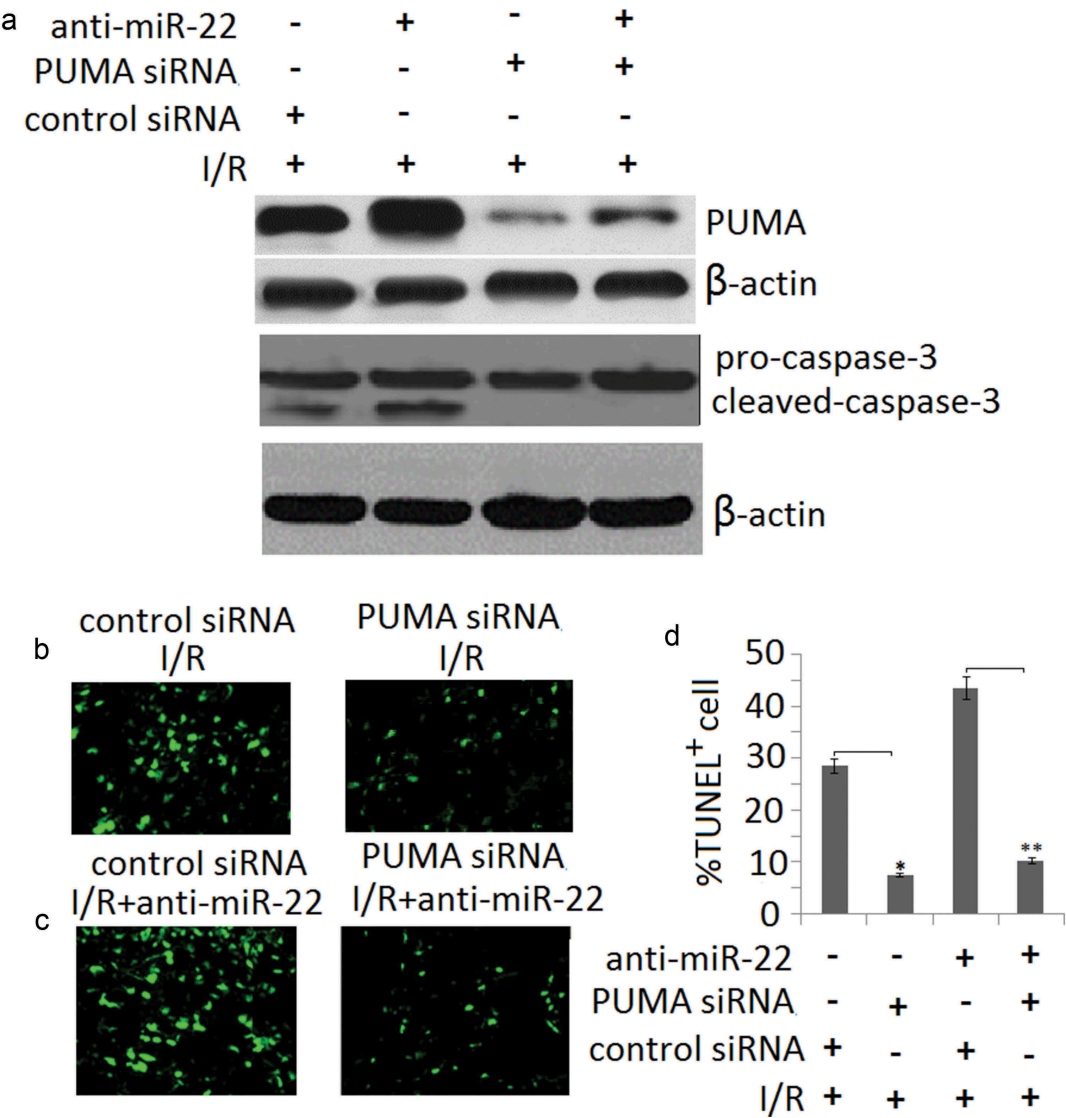
miR-22 inhibits cell apoptosis via targeting PUMA. PC12 cells were transfected with PUMA siRNA or/and anti-miR-22 or its controls then subjected to I/R as the methods in the ‘Materials and methods’ section. (a) PUMA and cleaved-caspase-3 protein expression was detected by Western blot assay; (b, c) Representative TUNEL staining in control and I/R groups; (d) The quantitative analysis of cell apoptosis in control and I/R groups, *p < 0.05; **p < 0.01.

To further analyze the contribution of miR-22 targeting PUMA to the biological function of miR-22 in PC12 subjected to I/R, we determined siRNA-mediated inhibition of PUMA protein expression. Western blot assay showed PUMA expression was blocked in PUMA siRNA transfected PC12 cells (Figure 5(a)). Cleaved-caspase-3 was also blocked in PUMA siRNA transfected PC12 cells (Figure 5(a)). As shown in Figure 5(b–d), following I/R or/and anti- miR-22 treatment, targeting PUMA significantly block anti-miR-22 or I/R-induced cell apoptosis compared to the control siRNA groups by TUNEL staining.

## Discussion

In this study, we report a role for miR-22 in mediating PUMA pro-apoptotic signaling in PC12 cells following I/R injury. We also demonstrate that the miR-22 is ischemic stress-responsive protectors against PC12 apoptosis. PC12 lacking miR-22 has an increased sensitivity to I/R-induced apoptosis, whereas PC12 overexpressing miR-22 has decreased sensitivity to I/R-induced apoptosis. Moreover, we identify that a molecular mechanism of PC12 protection by miR-22 is the inhibition of pro-apoptotic PUMA, which is target genes supported by our experimental evidences. Our findings suggest that the identified miR-22/PUMA may be involved in I/R-mediated neuroprotective signaling.

It was previously known that I/R injury downregulated miR-22 and overexpression of miR-22 protected cells from apoptosis upon brain [14], cardiac [11] and spinal cord ischemia [13], including ischemia/reperfusion-induced cerebral injury [15]. The cellular mechanism of such protection was investigated in rat and cell models. miR-22 was shown to modulate several inflammation-related genes (decreasing the activities of TNF-a, IL-6, COX-2, iNOS, Caspase-3, NCOA1 and NF-kB, while boosting IL-10 level) [11]. Previous publications have also shown that PUMA was upregulated by I/R injury in mouse small intestine [19] and cerebral astrocytes [20,21]. PUMA signaling could induce Caspase-3 cleavage and activation [19–21]. The information mentioned above was re-proven in our study (Figures 1, 3, 4 and 5(a))

It has previously shown that myocardial ischemia/reperfusion (MI/R) downregulated miR-22 expression. And enforced expression of miR-22 attenuated cardiomyocyte apoptosis and miR-22 target gene CREB [11]. However, Du et al. reported miR-22 level was significantly increased in rat hearts subjected to I/R injury [12]. H9c2 cardiomyocytes exposed to I/R also exhibited an increase of miR-22 expression. In addition, miR-22 inhibitor attenuated, whereas miR-22 mimic aggravated hypoxia/reoxygenation (H/R)-induced injury in H9c2 cardiomyocytes [12].

The clonal line PC12 originally derived from a solid rat adrenal medulla tumor has been widely characterized by the molecular, cellular, functional and stress studies to the point of being used to test validation of many compounds used in clinical therapy [25,26]. The ischemic-PC12 neuronal model [24,27] mimics in part stroke pathology since it is composed of a two-phase strategy; an OGD insult and a reperfusion (R) period which restored oxygen to the insulted tissue. Chen et al. reported that lncRNA ROR can promote the cerebral hypoxia/reoxygenation (H/R) injury in PC12 cells in vitro, a cellular model of cerebral ischemia/reperfusion injury, through inhibiting the expression of miR-135a-5p or upregulating the expression of ROCK1 and ROCK2 [28]. Zheng et al. reported that miR-130a expression was lower in PC12 cells after OGDR and in rats after MCAO. Moreover, ectopic-expression of miR-130a can significantly improve cell survival rate and reduce cell apoptosis and ROS production in PC12 cells after OGDR [29]. Liang et al. reported that OGD/R (I/R) treatment increased PC-12 cells’ injuries by miR-125b upregulation, and inhibition of miR-125b protects the PC-12 cells from OGD/R-induced injury by regulating the CK2α/NADPH oxidative signaling pathway [30]. Bioinformatics analysis confirmed miR-22 was continuously upregulated in a model of spinal cord following ischemia/reperfusion injury, but the role and the mechanism of miR-22 in spinal cord injury was unclear [13].

In the present study, we used PC12 as the I/R model *in vitro*. We demonstrated that the expression of miR-22 was much higher in the untreated PC12 cells. After I/R, the expression of miR-22 was significantly decreased in the PC12 cells, followed by increased cell apoptosis. Gain-of-function and loss-of-function approaches were used to investigate the therapeutic potential of miR-22 in PC12 subjected to I/R. The overexpression of miR-22 decreased

I/R-induced cell apoptosis in PC12 cells, and targeting miR-22 increased I/R-induced cell apoptosis in PC12 cells, suggesting that miR-22 may be a potential therapeutic approach against apoptosis for the clinical treatment of cerebral I/R.

Previous studies have reported that under ‘ischemic’ conditions, the stressed PC12 cells exhibit signs of apoptotic cell death as emphasized by a decrease of the Bax protein in the cytosol, release of cytochrome C from the mitochondria, activation of caspase-3, reduction of cyclin D1 and an increased DNA fragmentation [31–33]. It has recently found that PUMA is activated by oxidative stress in response to I/R to promote p53 independent apoptosis in the small intestine through the mitochondrial pathway. Inhibition of PUMA is potentially useful for protecting against I/R-induced intestinal injury and apoptosis [19]. Li et al. reported that inhibition of aberrant p53-PUMA feedback loop activation by intrathecal treatment with si-PUMA and PFT-α prevented IR-induced neuroapoptosis, inflammatory responses and BSCB breakdown by inactivating caspase 3-mediated apoptosis and NF-κB-mediated cytokine release [34]. Chen et al. reported that knockdown of PUMA led to the depressed expression of Bax, cleaved caspase-9 and caspase-3 during OSGD/R, indicating that PUMA is involved in the apoptosis of cerebral astrocytes upon I/R injury [35]. Our present study shows a novel finding that PUMA is regulated by miR-22 in PC12 cells. Moreover, we report for the first time that PUMA is a target of miR-22. Our findings suggest that inhibition of PUMA could be therapeutically beneficial for cerebral disease. Given that the PUMA apoptotic gene is target of miR-22 and are upregulated in ischemic conditions, patients with cerebral decrease with reduced levels of circulating miR-22 could be particularly suitable for future targeted treatments based on PUMA.

## Conclusion

In conclusion, miR-22 was downregulated and PUMA was upregulated in PC12 cells subject to I/R injury. Enforced miR-22 overexpression reduced the apoptotic rate of PC12 cells via inhibiting PUMA expression. Therefore, the miR-22/PUMA signals may provide a new insight to develop an effective pharmacological or genetic agent aimed at the molecular target.

## Highlights

1. miR-22 was downregulated in PC12 cells subject to I/R
2. PUMA was upregulated in PC12 cells subject to I/R
3. Enforced miR-22 overexpression reduced the apoptotic rate of PC12 cells
4. miR-22 overexpression reduced cell apoptosis in PC12 cells via downregulation PUMA

## References

[1] Sun J, Li YZ, Ding YH, et al. Neuroprotective effects of gallic acid against hypoxia/reoxygenation-induced mitochondrial dysfunctions in vitro and cerebral ischemia/reperfusion injury in vivo. Brain Res. 2014;1589:126–139. 25251593 10.1016/j.brainres.2014.09.039

[2] Chen L, Ren Z, Wei X, et al. Losartan protects against cerebral ischemia/reperfusion-induced apoptosis through ß-arrestin1-mediated phosphorylation of Akt. Eur J Pharmacol. 2017;815:98–108. 28844872 10.1016/j.ejphar.2017.08.028

[3] Li P, Shen M, Gao F, et al. An antagomir to microRNA-106b-5p ameliorates cerebral ischemia and reperfusion injury in rats via inhibiting apoptosis and oxidative stress. Mol Neurobiol. 2017;54:2901–2921. 27023223 10.1007/s12035-016-9842-1

[4] Macfarlane LA, Murphy PR. microRNA: biogenesis, function and role in cancer. Curr Genomics. 2010;11:537–561. 21532838 10.2174/138920210793175895 PMC3048316

[5] Carthew RW, Sontheimer EJ. Origins and mechanisms of miRNAs and siRNAs. Cell. 2009;136:642–655. 19239886 10.1016/j.cell.2009.01.035 PMC2675692

[6] Sempere LF, Freemantle S, Pitha-Rowe I, et al. Expression profiling of mammalian microRNAs uncovers a subset of brain-expressed microRNAs with possible roles in murine and human neuronal differentiation. Genome Biol. 2004;5:R13. 15003116 10.1186/gb-2004-5-3-r13 PMC395763

[7] Saraiva C, Esteves M, Bernardino L. microRNA: basic concepts and implications for regeneration and repair of neurodegenerative diseases. Biochem Pharmacol. 2017;141:118–131. 28709951 10.1016/j.bcp.2017.07.008

[8] Li WA, Efendizade A, Ding Y. The role of microRNA in neuronal inflammation and survival in the post ischemic brain: a review. Neurol Res. 2017;1–9. 10.1080/01616412.2017.1327505 28552032

[9] Li L, Stary CM. Targeting glial mitochondrial function for protection from cerebral ischemia: relevance, mechanisms, and the role of microRNAs. Oxid Med Cell Longev. 2016;2016:6032306. 27777645 10.1155/2016/6032306 PMC5061974

[10] Volný O, Kašicková L, Coufalová D, et al. microRNAs in cerebrovascular disease. Adv Exp Med Biol. 2015;888:155–195. 26663183 10.1007/978-3-319-22671-2_9

[11] Yang J, Chen L, Yang J, et al. microRNA-22 targeting CBP protects against myocardial ischemia-reperfusion injury through anti-apoptosis in rats. Mol Biol Rep. 2014;41:555–561. 24338162 10.1007/s11033-013-2891-x

[12] Du JK, Cong BH, Yu Q, et al. Upregulation of microRNA-22 contributes to myocardial ischemia-reperfusion injury by interfering with the mitochondrial function. Free Radic Biol Med. 2016;96:406–417. 27174562 10.1016/j.freeradbiomed.2016.05.006

[13] Li JA, Zan CF, Xia P, et al. Key genes expressed in different stages of spinal cord ischemia/reperfusion injury. Neural Regen Res. 2016;11:1824–1829. 28123428 10.4103/1673-5374.194754 PMC5204240

[14] Wang X, Shi C, Pan H, et al. microRNA-22 exerts its neuroprotective and angiogenic functions via regulating PI3K/Akt signaling pathway in cerebral ischemia-reperfusion rats. J Neural Transm (Vienna). 2020;127:35–44. 31883035 10.1007/s00702-019-02124-7

[15] Yu H, Wu M, Zhao P, et al. Neuroprotective effects of viral overexpression of microRNA-22 in rat and cell models of cerebral ischemia-reperfusion injury. J Cell Biochem. 2015;116:233–241. 25186498 10.1002/jcb.24960

[16] Hikisz P, Kilianska ZM. PUMA, a critical mediator of cell death–one decade on from its discovery. Cell Mol Biol Lett. 2012;17:646–669. 23001513 10.2478/s11658-012-0032-5 PMC6275950

[17] Chen L, Zhang D, Yu L, et al. Targeting MIAT reduces apoptosis of cardiomyocytes after ischemia/reperfusion injury. Bioengineered. 2019;10:121–132. 30971184 10.1080/21655979.2019.1605812 PMC6527071

[18] Toth A, Jeffers JR, Nickson P, et al. Targeted deletion of Puma attenuates cardiomyocyte death and improves cardiac function during ischemia-reperfusion. Am J Physiol Heart Circ Physiol. 2006;291:H52–60. 16399862 10.1152/ajpheart.01046.2005

[19] Wu B, Qiu W, Wang P, et al. p53 independent induction of PUMA mediates intestinal apoptosis in response to ischaemia-reperfusion. Gut. 2007;56:645–654. 17127703 10.1136/gut.2006.101683 PMC1942137

[20] Wang JK, Guo Q, Zhang XW, et al. Aglaia odorata Lour. extract inhibit ischemic neuronal injury potentially via suppressing p53/Puma-mediated mitochondrial apoptosis pathway. J Ethnopharmacol. 2020;248:112336. 31669102 10.1016/j.jep.2019.112336

[21] Ouyang YB, Xu L, Lu Y, et al. Astrocyte-enriched miR-29a targets PUMA and reduces neuronal vulnerability to forebrain ischemia. Glia. 2013;61:1784–1794. 24038396 10.1002/glia.22556 PMC3810393

[22] Zhang S, Zhang D, Yi C, et al. microRNA-22 functions as a tumor suppressor by targeting SIRT1 in renal cell carcinoma. Oncol Rep. 2016;35:559–567. 26499759 10.3892/or.2015.4333

[23] Aurora AB, Mahmoud AI, Luo X, et al. microRNA-214 protects the mouse heart from ischemic injury by controlling Ca (2)(+) overload and cell death. J Clin Invest. 2012;122:1222–1232. 22426211 10.1172/JCI59327 PMC3314458

[24] Tabakman R, Jiang H, Shahar I, et al. Neuroprotection by NGF in the PC12 in vitro OGD model: involvement of mitogen-activated protein kinases and gene expression. Ann N Y Acad Sci. 2005;1053:84–96. 16179511 10.1196/annals.1344.008

[25] Greene LA, Tischler AS. Establishment of a noradrenergic clonal line of rat adrenal pheochromocytoma cells which respond to nerve growth factor. Proc Natl Acad Sci U S A. 1976;73:2424–2428. 1065897 10.1073/pnas.73.7.2424 PMC430592

[26] Vaudry D, Stork PJ, Lazarovici P, et al. Signaling pathways for PC12 cell differentiation: making the right connections. Science. 2002;296:1648–1649. 12040181 10.1126/science.1071552

[27] Tabakman R, Jiang H, Levine RA, et al. Apoptotic characteristics of cell death and the neuroprotective effect of homocarnosine on pheochromocytoma PC12 cells exposed to ischemia. J Neurosci Res. 2004;75:499–507. 14743433 10.1002/jnr.20008

[28] Chen H, Li X. LncRNA ROR is involved in cerebral hypoxia/reoxygenation-induced injury in PC12 cells via regulating miR-135a-5p/ROCK1/2. Am J Transl Res. 2019;11:6145–6158. 31632583 PMC6789264

[29] Zheng T, Shi Y, Zhang J, et al. MiR-130a exerts neuroprotective effects against ischemic stroke through PTEN/PI3K/AKT pathway. Biomed Pharmacother. 2019;117:109117. 31226635 10.1016/j.biopha.2019.109117

[30] Liang Y, Xu J, Wang Y, et al. Inhibition of miRNA-125b decreases cerebral ischemia/reperfusion injury by targeting CK2a/NADPH oxidase signaling. Cell Physiol Biochem. 2018;45:1818–1826. 29510389 10.1159/000487873

[31] Dhivya Bharathi M, Justin-Thenmozhi A, Manivasagam T, et al. Amelioration of aluminum maltolate-induced inflammation and endoplasmic reticulum stress-mediated apoptosis by tannoid principles of emblica officinalis in neuronal cellular model. Neurotox Res. 2019;35:318–330. 30242626 10.1007/s12640-018-9956-5

[32] Zhong S, Zhu J, Li Y, et al. Butylene fipronil induces apoptosis in PC12 murine nervous cells via activation of p16-CDK4/6-cyclin D1 and mitochondrial apoptotic pathway. J Biochem Mol Toxicol. 2019;33:e22264. 30597675 10.1002/jbt.22264

[33] Wani WY, Kandimalla RJL, Sharma DR, et al. Cell cycle activation in p21 dependent pathway: an alternative mechanism of organophosphate induced dopaminergic neurodegeneration. Biochim Biophys Acta Mol Basis Dis. 2017;1863:1858–1866. 27262357 10.1016/j.bbadis.2016.05.014

[34] Li XQ, Yu Q, Chen FS, et al. Inhibiting aberrant p53-PUMA feedback loop activation attenuates ischaemia reperfusion-induced neuroapoptosis and neuroinflammation in rats by downregulating caspase 3 and the NF-?B cytokine pathway. J Neuroinflammation. 2018;15:250. 30172256 10.1186/s12974-018-1271-9 PMC6119253

[35] Chen H, Tian M, Jin L, et al. PUMA is involved in ischemia/reperfusion-induced apoptosis of mouse cerebral astrocytes. Neuroscience. 2015;284:824–832. 25451294 10.1016/j.neuroscience.2014.10.059

